# Examining modal and amodal language processing in proficient bilinguals: evidence from the modality-switch paradigm

**DOI:** 10.3389/fnhum.2024.1426093

**Published:** 2024-10-08

**Authors:** Dirk Wentura, Elisabeth Shi, Juliane Degner

**Affiliations:** ^1^Department of Psychology, Saarland University, Saarbrücken, Germany; ^2^Department of Psychology, University of Hamburg, Hamburg, Germany

**Keywords:** bilingualism, embodiment, modal processing, amodal processing, switch costs

## Abstract

Recent discussions have emphasized the significance of embodied processing in language comprehension. Nevertheless, continuous debates persist regarding the relative contribution of modal (embodied) and amodal (abstract) processing of language. The current study investigated the contribution of modal processing in the first (L1) and second (L2) language, hypothesizing higher level of abstract amodal symbol processing in L2 than L1, since the correspondence of L1 and L2 (i.e., the symbol-to-symbol assignment) is in the foreground when learning L2. We employed the modality-switch paradigm (Pecher et al., 2003) in both German and French versions with proficient sequential German and French bilinguals (*N* = 79). Participants were presented with noun-adjective pairs (e.g., keys – jingling) in both languages and decided whether the adjective could be applied to the noun. This task repeatedly requires switching modality between trials, (e.g., from auditory [keys – jingling] to olfactory [soap – perfumed]), typically causing switch costs on response latency as compared to maintaining the modality. Contrary to the hypothesis, we observed modality switch effects (MSE) in both L1 and L2. This result suggests that embodied language processing occurs not only in the first language but also extends to the second language thus challenging the assumption that L2 processing predominantly involves abstract amodal symbol processing. Notably, however, significant L1 and L2 MSEs were found for French, whereas for German already the L1 effect was rather weak (though significant); the corresponding L2 effect was not significant. Thus, the results hinted at differences between languages regarding the relative role of modal and amodal processing.

## 1 Introduction

In cognitive psychology, a longstanding debate revolves around the format of mental representations (e.g., Barsalou, 1999; Kaup et al., 2024). Early perspectives emphasized amodal abstract symbolic representational formats (e.g., Bransford and Franks, 1971; Collins and Loftus, 1975; Fodor, 1975; Pylyshyn, 1984) to account for the adaptability and flexibility of language and thought processes. Over the past decades, however, researchers highlighted limitations of a purely amodal representation and postulated embodiment as the foundation of conceptual knowledge, grounded in sensorimotor systems (Barsalou, 1999, 2010). According to this perspective, processing a mental concept involves the reenactment of embodied perceptual and motor processes.

The basic idea of this approach is best illustrated by the study of Pecher et al. (2003), who made a strong empirical case for embodied processing of language by introducing the property verification task with a modality-switch design. They presented participants with noun-adjective pairs (e.g., *keys* – *jingling*) and instructed them to decide as quickly as possible (while maintaining a high level of accuracy) whether the adjectives were applicable to the nouns. (Of course, the task included filler trials with non-applicable pairs [e.g., *lawn* – *proud*] to make it meaningful). Adjectives in relevant trials always addressed a specific modality (i.e., the auditory modality in the example). Unknown to the participants, the experimental procedure included systematic sequences of double trials in which the first prime trial adjective addressed either the same or different modality as the second probe trial adjective. For example, the probe trial “*keys – jingling*” focusing on the auditory modality would be preceded by either a same-modality prime trial “*bees – buzzing*” or a different modality prime trial “*salsa – spicy*.” The authors convincingly argued that if the verification of attributes involves modality-specific processing, switching modality between prime and probe trials would cause processing costs relative to modality maintenance. If, on the other hand, verification was a process in an abstract amodal knowledge system, such manipulation should have no effect on response speed or accuracy. They indeed observed reliable switching costs of in two experiments, a result that was replicated in several other studies (Marques, 2006; Pecher et al., 2004; Scerrati et al., 2015; Scerrati et al., 2017; van Dantzig et al., 2008; Vermeulen et al., 2007).

A more integrative view would emphasize that there is no exclusive type of mental representation, but that modal and amodal forms coexist and have different functional roles (Kaup et al., 2024; Martin, 2007). For example, it has been argued that modal processing of language is complemented by amodal processing as in the hub-and-spoke model of neurocognition (Lambon Ralph et al., 2010; Patterson et al., 2007; Pobric et al., 2010), in which a concept is represented by modality-specific codes (the “spokes”) that are integrated into an amodal “hub” structure (see also Martin, 2007).

### 1.1 Embodied cognition and second language processing

An extremely interesting case in this regard is the (proficient) use of a second language (L2), especially when L2 is learned later than a person’s first language (L1) as in sequential bilingualism. The acquisition of L1 *per se* can be considered a plausible argument in favor of an embodiment rationale, since the early acquisition of L1 is dominated by direct perceptual experiences. The later acquisition of L2, can be argued to occur primarily through the transfer of L1 concepts, especially when it occurs during formal schooling rather than direct experiences (e.g., Altarriba, 2003; Harris, 2004). L2 representation may thus occur rather at the level of amodal symbol systems, thus lack embodiment. A similar argument has been brought forward with regard to the reduced emotionality of L2 experiences (Degner et al., 2012; Opitz and Degner, 2012). Based on this rationale, one might speculate that L2 processing is not accompanied by the same sensorimotor processes that have been demonstrated for L1. Thus, thinking in L2 may involve less embodied cognition than thinking in L1—at least for sequential bilinguals.

Moreover, if sensorimotor processes were absent in the processing of a second language, what implications does this hold for their role in language processing overall in general? Are these processes non-obligatory, perhaps even lacking functional relevance? Conversely, if L2 processing exhibits similar embodiment phenomena as observed in L1, despite differences in acquisition, could this be construed as evidence supporting the mandatory involvement of sensorimotor processes in language processing?

Recently, Kühne and Gianelli (2019) reviewed studies investigating L1–L2 differences in terms of embodiment and grounding. They conclude that the evidence is still limited. We will give a brief outline of this research.

Dudschig et al. (2014) sampled Germans proficient in English as L2, who completed a hybrid of the Stroop task (Stroop, 1935) and the action compatibility task (Glenberg and Kaschak, 2002): Participants were instructed to categorize the color of word stimuli by either an upward or downward action (i.e., releasing a button press in the middle of a vertical response panel and pressing a button at the top or bottom of the panel). Although word meaning was task-irrelevant, words associated with a typical up/down location (e.g., star, root) produced an action compatibility effect, i.e., up responses (e.g., to star) and down responses (e.g., to root) were faster than the reverse assignments. Notably, this compatibility effect occurred in both L1 and L2.

Buccino et al. (2017) found motor system involvement in the processing of graspable objects for Italian participants with English as L2. In a go/no-go task, participants had to press a key when a word (as opposed to pseudowords) or a meaningful picture (as opposed to scrambled pictures) was shown. Responses were slower when words or pictures referred to objects that could be grasped (as opposed to objects that could not be grasped), suggesting involuntary involvement of the motor system. A similar effect in L1 has already been shown by (Marino et al. 2014).^1^

Vukovic and Shtyrov (2014) tested German participants with English as L2, presenting L1 and L2 action verbs in a passive word-reading paradigm accompanied by EEG measurements. They tested the activity of the cortical motor system using a specific index derived from the EEG data (i.e., event-related desynchronization of the mu rhythm) and observed evidence for motor system activation in L1 and L2; however, L2 activity seemed to be lower than L1 activity.

In a recent study by Norman and Peleg (2022) participants had to check whether a pictured object was mentioned in a preceding sentence. To test for visual simulation, in critical trials the picture presented the object in the implied form (e.g., a picture of a lemon slice following “The girl saw the lemon in the tea.”; match trials) or not (i.e., a picture of a whole lemon; non-match trials). Response time differences between match and non-match trials (indicating the degree of congruence of the visual simulation with the factual picture) were found only for L1 in a sample of Hebrew L1 participants; the effect was not found for these participants in English (L2).

In addition to behavorial and EEG experiments, which typically assess rapid processes triggered by individual stimuli, several fMRI studies have been conducted. For example, De Grauwe et al. (2014) presented Dutch (L1) and bilingual German-Dutch (L2) participants with Dutch motor-related and non-motor-related verbs and found higher activation for motor than for non-motor verbs in motor and somatosensory brain areas in L1 and L2 (see also Zhang et al., 2020). Recently, Monaco et al. (2023) replicated this finding by testing samples of German and French participants (with French and German as L2, respectively) in a silent reading task with blocks comprising always eight verbs in a 2 (motor-related vs. non-motor-related) × 2 (L1 vs. L2) design. For blocks of motor-related verbs (compared to non-motor-related verbs), they found stronger activation of motor and premotor areas with no discernible moderation by language (L1 vs. L2). Somewhat surprisingly, there was also a main effect of language with motor and premotor areas being more strongly activated during L2 (vs. L1) blocks, regardless of verb modality.

In summary, there is some evidence for embodiment processes in L2. However, the few available studies have focused almost exclusively on the action/motor aspect of embodiment. A recent study by Norman and Peleg (2022) which focused on visual simulation found no evidence for this in L2. In addition, all of the previous studies reported above—with the exception of the recent fMRI study by Monaco et al. (2023)—lacked a full cross-over design that contrasts two subsamples for whom the involved languages were L1 or L2, thus confounding the L1 vs. L2 contrast with the actual language used for instructions and experimental stimuli.

Surprisingly, the property verification task with a modality switch design (Pecher et al., 2003), as introduced above, has not yet been used for investigating L2 embodiment. For the sake of transparency, Zhao et al. (2020) recently used a variant of the modality switch design with Chinese-English bilinguals. However, instead of the property verification task, they used lexical decision tasks (Experiments 1 and 2) and a naming task (Experiment 3) with single words as targets; the experimental trials were double trials in which both words matched in modality (i.e., visual–visual, auditory–auditory) or did not match (i.e., visual–auditory, auditory–visual). That is, they tested whether the accessibility of words was increased when the preceding word addressed the same modality as compared to a different modality. They did find some evidence for modality switching effects in L2. However, this result should be taken as preliminary, since the effect may also be explained with semantic priming processes (e.g., McNamara, 2013): Words of the same modality can also be associated in an amodal representational system (see already Pecher et al., 2003, for a discussion of this problem); lexical decision and word naming are the standard tasks in semantic priming research (e.g., McNamara, 2013).

### 1.2 The present study

In our experiment, we applied the modality switch paradigm in a German and a French language version to samples of German-French and French-German sequential bilinguals. By employing this full cross-over design consisting of two different L1–L2 samples, we avoid potential confounding of the L1 vs. L2 contrast with the contrast of the actual languages.

Furthermore, to ensure that a possible L1/L2 moderation of the modality switching effect is not due to a potential general performance deficit in L2, we added a control task. As in Degner et al. (2012), we used a sequential semantic priming task (see Neely, 1991; McNamara, 2005, 2013) to assess automatic semantic language processing in L1 and L2.

## 2 Method

### 2.1 Participants

Recruitment focused on inviting sequential and unbalanced bilinguals with proficient knowledge of L2.^2^ Seventy-nine bilingual students were recruited at University of Saarland, Saarbrücken, a South-west German university bordering France. Thereof 42 were German-French bilinguals (7 men, 35 women; age range 19–35 years, *Md* = 23 years) and 37 were French-German bilinguals (10 men, 27 women; age range 15–26 years,^3^ *Md* = 21 years). Data of two additional French-German bilinguals were discarded because of low L2 proficiency—they knew less than 70% of the L2 words used in the modality-switch task (see *Procedure*).

We determined the sample size targeting an average modality switch effects in prior research. The effect in four different experiments ranged from *d_Z_* = 0.27–0.37 with an average of *d_Z_* = 0.32 (Pecher et al., 2003; Marques, 2006; van Dantzig et al., 2008). Detecting an effect of *d_Z_* = 0.30 with power 1 − *β* = 0.80 (*α* = 0.05, one-tailed), a sample of *N* = 71 is required. Factual power with *N* = 79 was 1 − *β* = 0.84.

In order to characterize the sample, participants completed a language history questionnaire (Degner et al., 2012) at the end of the procedure, containing questions on age and context of L2 acquisition, as well as duration, and purpose of stay(s) abroad. Participants rated their L2 proficiency referring to different aspects of language use (vocabulary, accent, comprehension, writing, reading, overall) using a 10 cm long line as scale with the anchors zero (none) and 100 (native language-like). The five aspects were highly correlated (Cronbach’s alpha = 0.91) and thus combined to a single variable *self-rated proficiency*. Furthermore, participants rated the present-day frequency of L2 use in daily live on seven-point scales (1 = *not at all*, 7 = *exclusively*), separated for three domains: professional (study, job, textbooks), private-personal (family, friends, partner), and private-leisure (fiction, TV, movies, radio). We averaged the three domains to one variable *frequency of language use*. Table 1 shows detailed descriptive statistics for both samples. According to these self-reports in the language history questionnaire, all included participants can be regarded as sequential and unbalanced bilinguals with proficient knowledge of L2.

**Table 1:** Mean values (standard deviations in parentheses) and group comparisons of language history responses for both samples.

	French (*n* = 37)		German (*n* = 42)	*t*		*p*
Age of begin of L2 acquisition (year)	8.2	(5.1)	10.9	(3.7)	2.73^a^	0.008
Duration of longest stay (months)^b^	12.0	(8.0–19.0)	10.0	(6.0–12.0)	0.12	0.902
Sum duration of all stays (months)^b^	21.5	(14.5–48.0)	13.0	(7.0–24.0)	1.69	0.097
Self-rated L2 proficiency (0–100)	68.5	(16.3)	71.4	(14.3)	0.82	0.414
Intensity of L1 use (1–7)	5.0	(1.1)	5.8	(0.7)	3.98^a^	<0.001
Intensity of L2 use (1–7)	4.0	(0.7)	3.6	(1.2)	1.73^a^	0.089

a t-test for unequal variances.

b To account for skewed distribution, median (instead of mean) and interquartile range (instead of SD) are reported; t-test was computed on log-transformed variables.

### 2.2 Design

For the modality switch task, a 2 (stimulus language: French vs. German) by 2 (trial type: modality switch vs. non-switch) repeated measures design was implemented. For the semantic priming task, a 2 (Stimulus language: French vs. German) by 2 (Prime-target relation: semantically related vs. non-related) repeated measures design was implemented. Additionally, the order of task language (French first vs. German first) and the assignment to one of two complementary stimulus sets (see *Materials*) were counterbalanced between subjects.

### 2.3 Materials

For the modality-switch task, we essentially used the stimulus materials of Pecher et al. (2003), that is, a set of 100 critical concept-property items and 200 filler items. Words were first translated from English to German and then from German to French. In few cases, we deviated from direct translations by creating new concept-property items, for example, if translations of different English adjectives led to the same German or French adjective.^4^ Whenever an item was exchanged, modality was preserved. That is, as Pecher et al. (2003), for the critical items we used 26 properties from vision, 24 from motor actions, 18 from audition, 12 from touch, 12 from taste, and 8 from smell. Pecher et al. (2003, p. 120) justified this imbalance arguing that “some modalities have more words for properties than others, [therefore] the number of properties differed across modalities by necessity.” We had no reason to question this and also found it sensible to use the same material.

The critical items were arranged to pairs—*context trial* followed by *target trial*—such that 25 pairs include a modality switch (*switch trials*) and 25 were of same modality (*no switch trials*). There were two lists (A and B) of these items: If a given item was part of a switch trial in List A, it was part of a no-switch trial in List B. The two lists were used in a counterbalanced design. In order to balance the rate of true and false items, filler item pairs were included of which 50 consisted of two false items and 50 consisted of a true item followed by a false item (see Pecher et al., 2003).

For the semantic priming task, we used the same materials as Degner et al. (2012), that is, 50 semantically related word pairs (e.g., PLAGE – SABLE [beach – sand]) were selected from published studies on semantic priming in French (Grainger and Beauvillain, 1988; Isel and Bacri, 1999; New et al., 2004) and translated into German (e.g., STRAND – SAND). It was assured that French and German equivalents matched according to word length and frequency of use. Semantically unrelated word pairs were formed by randomly reassigning the target words of the stimulus list to the prime words. Nonword targets were constructed by exchanging one letter in each target word (e.g., SABLE in SUBLE, STRAND in STRUND).

To avoid presentation of translation equivalents within the French and the German version of each task, all stimulus lists were divided into two subsets that matched according to valence, frequency, and word length. Thus, if participants received stimulus set A in the French version of the task, they received stimulus set B in the German version to ensure that repeated presentation of the stimuli and their translation equivalents would not bias results.

### 2.4 Procedure

The experiment was conducted in groups of up to five participants at separated personal computers using EPrime 2.0 for the modality switch task and INQUISIT 1.33 for the semantic priming task. Introductions were presented in both languages, French and German, on the computer screen. Specific task instructions were given in the language of the respective experimental block.

Participants first completed the modality switch tasks. Participants completed the task twice, once in French and once in German with counterbalanced order of language. They were instructed to quickly decide whether the property can be considered to be true for the respective concept. At the beginning of each language block, participants first worked through a brief practice phase comprising six trials. Each main block started with two warm-up trials that did not enter into the analysis, followed by 300 trials (i.e., 150 trial pairs). After every 50 pairs, the participants could take a short break. Each trial started with the presentation of a fixation stimulus (“*****”) in the middle of the screen that was replaced after 500 ms by a noun/property pair,^5^ which remained on screen until a response was recorded. Participants were instructed to press the ‘D’ key if the property can be used to describe the noun, the ‘K’ key if not. They were instructed to respond as fast and accurately as possible. If they pressed the wrong key, an error message appeared on screen for 600 ms; if no response was given within 2,000 ms, a “too slow” message appeared (again for 600 ms). The next trial started after an interval of 1,000 ms.

After completing the modality switch task, participants received a list of all L2 words in alphabetical order that were presented to them during the (L2) property verification task (i.e., all nouns and all properties). They were instructed to “tick the words whose meaning was not clear to [them] … in the experiment and which [they] …would look up in a dictionary if necessary.”

Subsequently, the semantic priming tasks were administered. These were introduced as lexical processing tasks. Participants were informed that word pairs would again be presented on the screen in rapid succession, that they should ignore the first word (the prime) and categorize the second word (the target) as quickly as possible as a word or non-word. As response keys were used: [5] = word, [A] = nonword. Each trial began with the presentation of a fixation cross in the center of the screen, which was replaced by the prime word after 200 ms. The prime remained on the screen for 150 ms and was immediately overwritten by the target word (i.e., stimulus onset asynchrony = 150 ms), which remained on the screen until a response was recorded. Participants were instructed to respond as quickly and accurately as possible. If they pressed the wrong key, an error message appeared on the screen for 200 ms. The next trial began after an interval of 1,000 ms. Each semantic priming task consisted of a random sequence of 100 trials in which each prime word was presented four times; once with its related target, once with an unrelated target, once with the nonword derived from the related target, and once with a nonword derived from an unrelated target. For each participant, the order of language was the same as in the modality switch task. After the priming tasks, participants completed the language history questionnaire.

## 3 Results

We report all measures, manipulations and exclusions for our studies. All data are openly accessible at https://osf.io/anzr3. The criterion of significance was set to *α* = 0.05. In accordance with our power planning, tests for simple semantic priming effects and simple modality switch effects were one-tailed (in case of a positive effect, which applies to all reported cases, except one; see below).

We applied linear mixed model analyses (lmm). We used the functions lmer() for RTs and glmer(… family = “binomial”) for accuracies of the package lme4 (Bates et al., 2015) of the R environment for statistical computing (R-Core-Team, 2016). Moreover, we used the lmerTest package (Kuznetsova et al., 2016), which allows estimation of degrees of freedom (using Satterthwaite’s approximation) and thus *p*-values for the tests of regression weights.

We report effect sizes according to Westfall et al. (2014; see also Brysbaert and Stevens, 2018). We added the report of *d_Z_* values whenever simple priming or modality switch effects are reported. The *d_Z_* values are based on variables aggregated across trials per participant. They can be directly used for meta-analyses or for power planning of experiments that are comparable in the number of trials to the present tasks.

### 3.1 Modality switch task

We removed RTs of trials in which an error occurred as well as RTs of trials with an error in the context trial (i.e., the preceding trial). With this procedure, we followed the approach by Pecher et al. (2003) who argued that reliably measuring modality switch effects depends on participants correctly processing the modality in both trials. The relative number of valid trials for the RT analysis was thus reduced to 70.0% for L1 and 50.9% for L2. Moreover, trials with outlying response latencies above 1.5 interquartile ranges above the third quartile with respect to the individual RT distribution of the given task (i.e., L1 and L2; see Tukey, 1977) or below 300 ms were considered invalid and excluded from further analyses (1.5 and 1.4% of all L1 and L2 trials, respectively). Means and standard errors for both modality switch tasks are reported in Table 2.

**Table 2:** Mean RT (in ms) and accuracies (%; in parentheses) of (A) the property verification task and (B) the semantic priming task as a function of modality-switch or semantic relation, respectively, between prime and target, stimulus language, and participants’ L1; priming effects/modality-switch effects (for RTs; standard errors in brackets).

	French participants				German participants					
(A) Property verification										
Task language	No-switch		Switch		No-switch		Switch		MS^a^	
L1	978	(82.9)	1,027	(77.6)	951	(91.9)	966	(91.1)	31	[9]
L2	1,174	(75.1)	1,179	(74.9)	1,165	(75.5)	1,201	(73.5)	21	[15]
(B) Semantic priming										
Task language	Related		Unrelated		Related		Unrelated		SP^b^	
L1	558	(97.5)	573	(96.7)	556	(94.0)	573	(92.0)	16	[3]
L2	649	(93.4)	674	(90.9)	607	(94.5)	636	(92.6)	28	[4]

a Modality-switch effect, RT_Switch_ – RT_No-switch_; standard error in brackets.

b Semantic priming effect, RT_Unrelated_ – RT_Related_; standard error in brackets.

#### 3.1.1 Response times

The fixed variables of our lmm model were modality (switch vs. no-switch), task language (L1 vs. L2), participant L1 (French vs. German), and their interaction terms with modality and task language as level 1 predictors and L1 as level 2 predictor. Since a model with full random slopes for items (i.e., modality × task language) did not converge, the following model structure was used:

$\begin{array}{l} RT\sim \mathrm{modality}\times \mathrm{task lang}.\times L1\\ +\left(1+\mathrm{modality}\times \mathrm{task lang}.|\;\mathrm{participants}\right)\\ +\left(1+\mathrm{modality}\;|\;\mathrm{item}\right) \end{array}$

Table 3 (top part) summarized the results of this analysis. There are three significant effects: Beside the trivial main effect of task language effect indicating that responses in L2 are slower than in L1, there is the expected significant main effect of modality switch: Responses are slower after a modality switch than after a modality repetition. Notably, this effect is not significantly moderated by task language (L1 vs. L2).

**Table 3:** Results of the linear mixed-model analyses for the modality switch task.

Fixed factor	Coefficient	SE	df	*t*	*p*	*d* ^a^
Main analysis						
*Intercept*	1,094.7	19.0	161.6	57.51	<0.001	
Mod. Switch (M)	13.0	3.9	63.9	3.34	0.001	0.089
Task Lang. (L1 vs. L2; T)	−102.7	9.4	76.4	−10.96	<0.001	0.703
L1	−9.1	13.8	76.9	−0.66	0.511	0.063
M × T	5.9	4.1	83.3	1.43	0.157	0.040
M × L1	−0.3	3.9	99.4	−0.08	0.934	0.002
T × L1	−20.5	16.1	152.7	−1.27	0.205	0.140
M × T × L1	−9.0	4.2	62.0	−2.15	0.035	0.062
French language						
*Intercept*	1,115.0	26.7	105.4	41.71	<0.001	
Mod. Switch (M)	22.5	6.2	36.6	3.65	<0.001	0.146
L1	93.3	18.3	75.8	5.11	<0.001	0.607
M × L1	−6.6	5.8	68.0	−1.14	0.259	0.043
German language						
*Intercept*	1,074.5	23.0	100.9	46.64	<0.001	
Mod. Switch (M)	3.6	4.9	74.4	0.73	0.467	0.026
L1	−111.7	15.0	76.9	−7.45	<0.001	0.807
M × L1	6.1	4.9	74.2	1.26	0.212	0.044

Coding of predictor variables was as follows: Mod. Switch (M): −1 = no switch, +1 = switch; Task Language (L1 vs. L2): −1 = L2, +1 = L1; Task Language (G vs. F): −1 = French, +1 = German; L1: −1 = French, +1 = German.

a *d* is the effect size according to Westfall et al. (2014; see also Brysbaert and Stevens, 2018), i.e., the mean difference between conditions (i.e., here the doubled coefficient due to the coding) divided by the square root of the sum of all random variances (i.e., of intercepts, slopes, residual).

**Figure 1:**
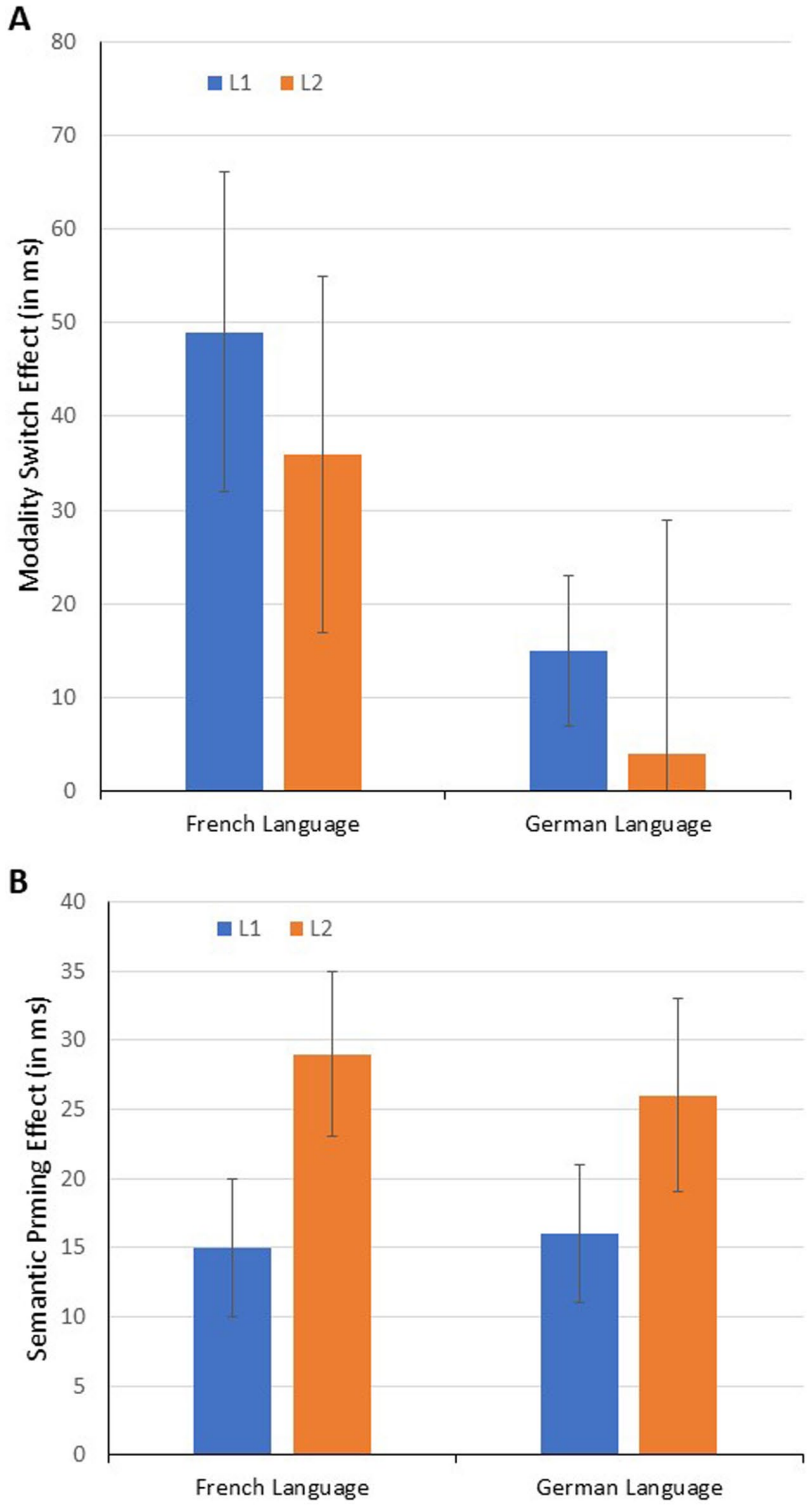
Modality switch effects **(A)** and semantic priming effects **(B)** as a function of language and L1/L2 status (i.e., the L1 bar for French language refers to the French sample, the corresponding L2 bar refers to the German sample; the L1 bar for German language refers to the German sample, the corresponding L2 bar refers to the French sample; the whiskers denote +1/−1 standard error based on variables aggregated across trials per participant).

The third significant effect is the triple interaction. Note that due to our Latin square design, the test for the triple interaction is in fact the test for general differences in the modality switch effect between German and French language, irrespective of whether it is a L1 or L2 effect (see Figure 1A). Therefore, to decompose this interaction, we conducted two follow-up analyses, one for the French language, one for the German language (Table 3, bottom part). There is a substantial modality switch effect in the French language, irrespective of whether French was L1 or L2. The effect was significant in L1 (i.e., for the French-German speakers), *t*(30.7) = 2.81, *p* = 0.004 (one-tailed), *d* = 0.20 (*d_Z_* = 0.47), as well as in L2 (i.e., for the German-French speakers), *t*(36.03) = 2.00, *p* = 0.027 (one-tailed), *d* = 0.10 (*d_Z_* = 0.30).

For the German language, the main effect of modality switch was not significant and there was no significant interaction with L1 sample (see Table 3). Closer inspection revealed that the modality switch with German language stimuli was significant in L1 (i.e., for German-French speakers), *t*(1,098.4) = 2.13, *p* = 0.017 (one-tailed), d = 0.08 (*d_Z_* = 0.27); for L2 (i.e., French-German speakers), the effect was numerically negative (but not significant in a two-tailed test, |*t*| < 1).

In anticipation of the discussion (see below), we additionally tested whether modality switch effects in L1 significantly differed between French and German; but the difference missed the criterion of significance, *t*(46.05) = 1.68, *p* = 0.099, *d* = 0.05 (*d* = 0.43).

Figure 1A shows the effects (means and standard errors based on variables aggregated across trials per participant).

On an exploratory note, we repeated the two main analyses by (a) additionally including block order (i.e., first block = L1 vs. first block = L2) with all interaction terms and (b) intensity of L2 use (*z*-standardized) with all interaction terms. In all four analyses, the significant terms involving modality switch reported above (in Table 3) were still significant in these control analyses; no further term involving modality switch was significant.

#### 3.1.2 Accuracies

As usual for RT-base paradigms (to potentially identify speed-accuracy tradeoffs), we additionally analyzed response accuracy corresponding to the main analysis of RTs, using the R function glmer (family = “binomial”).^6^ Again, we observed a significant main effect of task language, *β* = 0.447, *z* = 8.38, *p* < 0.001 indicating that the probability of a correct response was increased for L1 compared to L2. The main effect of L1 sample was significant, *β* = 0.244, *z* = 3.85, *p* < 0.001, and the task language × L1 interaction was associated with *β* = 0.221, *z* = 1.76, *p* = 0.078. As can be seen in Table 2, this pattern is dominantly due to the French sample being less accurate in their L1 than the German sample. The modality switch effect was not significant, *β* = −0.062, *z* = 1.18, *p* = 0.239; numerically, however, it conforms to the RT effect, that is, subsequent to a switch, the probability of an error is increased. Thus, there is no indication of a speed-accuracy tradeoff. All other effects were not significant, |*z*| < 1.22.

### 3.2 Semantic priming

We removed RTs of trials in which an error occurred (4.1 and 7.9% for L1 and L2, respectively). Moreover, trials with outlying response latencies above 1.5 interquartile ranges above the third quartile with respect to the individual RT distribution of the given task (i.e., L1 and L2; see Tukey, 1977) or below 300 ms were considered invalid and excluded from further analyses (4.4 and 4.1% for L1 and L2, respectively). There was a typo in one French target word of the lexical decision task (*balein* instead of *baleine*); these trials were discarded as well. Means and standard errors for both priming tasks are reported in Table 2.

#### 3.2.1 Response times

The fixed variables of our lmm model were relation (related vs. unrelated), task language (L1 vs. L2), participant L1 (French vs. German), and their interaction terms with relation and task language as level 1 predictors and L1 as level 2 predictor. A full random effect structure (see Barr et al., 2013) was used:

$\begin{array}{l} RT\sim \mathrm{relation}\times \mathrm{task lang}.\times L1\\ +\left(1+\mathrm{relation}\times \mathrm{task lang}.|\;\mathrm{participants}\right)\\ +\left(1+\mathrm{relation}\times \mathrm{task lang}.|\;\mathrm{items}\right) \end{array}$

Table 4 (top part) shows the results. The analysis revealed a trivial main effect of task language (i.e., responses in L2 are slower than L1 responses), which was more pronounced for the French sample as indicated by the significant interaction of task language and participant L1. Importantly, it yielded the expected significant semantic priming effect: Responses were faster for targets preceded by a related prime than for targets preceded by an unrelated prime. The semantic priming effect was not significantly moderated by task language; numerically, the effect was even stronger in L2 compared to L1. We tested both effects separately (see Table 4, middle and bottom parts). For L1 as well as for L2, we found robust semantic priming effects in both the French-German and German-French speakers, indicating sufficient L2 proficiency in both groups of participants.

**Table 4:** Results of the linear mixed-model analyses for the semantic priming task.

Fixed factor	Coefficient	SE	df	*t*	*p*	*d* ^a^
Main analysis (L1 vs. L2)						
*Intercept*	605.0	9.2	92.8	65.64	<0.001	
Relation (R)	−11.2	1.5	150.3	−7.56	<0.001	0.158
Task Lang. (L1 vs. L2; T)	−39.3	3.8	90.9	−10.26	<0.001	0.553
L1	−11.1	8.9	79.8	−1.26	0.212	0.157
R × T	3.0	1.8	69.4	1.70	0.094	0.042
R × L1	−0.8	1.6	92.8	−0.50	0.619	0.011
T × L1	10.3	4.6	136.4	2.23	0.028	0.145
R × T × L1	−0.2	1.7	79.0	−0.11	0.917	0.002
L1						
*Intercept*	565.7	9.2	88.9	61.48	<0.001	
Relation (R)	−8.0	1.9	89.4	−4.24	<0.001	0.125
L1	−1.0	9.2	88.9	−0.11	0.915	0.015
R × L1	−0.9	1.9	89.4	−0.49	0.625	0.014
L2						
*Intercept*	643.8	10.7	94.5	60.10	<0.001	
Relation (R)	−13.9	2.3	50.2	−6.07	<0.001	0.179
L1	−21.3	10.7	94.5	−1.99	0.050	0.274
R × L1	−0.8	2.3	50.2	−0.36	0.720	0.011

Coding of predictor variables was as follows: Relation (R): −1 = unrelated, +1 = related; Task Language (L1 vs. L2): −1 = L2, +1 = L1; L1: −1 = French, +1 = German.

a *d* is the effect size according to Westfall et al. (2014; see also Brysbaert and Stevens, 2018), i.e., the mean difference between conditions (i.e., here the doubled coefficient due to the coding) divided by the square root of the sum of all random variances (i.e., of intercepts, slopes, residual).

On an exploratory note, we repeated the main analyses by (a) additionally including block order (i.e., first block = L1 vs. first block = L2) with all interaction terms and (b) intensity of L2 use (*z*-standardized) with all interaction terms. In both analyses, the significant priming effect reported above (and in Table 4) was still significant in these control analyses; no further term involving the relation factor was significant, except one in the analysis including block order. In this analysis, the triple interaction relation × task lang. × block order was significant, *t*(77.35) = 2.01, *p* = 0.048. For the priming effect in L1, block order caused no difference, *t*(72.54) = 1.01, *p* = 0.317; the L1 priming effect (16 ms) was significant *t*(54.23) = 4.17, *p* < 0.001. For the priming effect in L2, however, block order caused a difference, *t*(70.28) = 2.23, *p* = 0.029. Those, who started with L1 had a larger L2 priming effect (38 ms) compared to those who started by L2 (18 ms); both priming effects, however, were significant, *t*(37.76) = 5.63, *p* < 0.001 (one-tailed) and *t*(76.47) = 2.89, *p* = 0.003 (one-tailed).

In summary, semantic priming effects for all cells of the 2 (language: French vs. German) × 2 (L1 vs. L2) conditions were significant with *t*(27.93) = 2.57, *p* = 0.008 (one-tailed), *d* = 0.11 (*d_Z_* = 0.48), for French (L1), *t*(40.65) = 5.10, *p* < 0.001 (one-tailed), *d* = 0.20 (*d_Z_* = 0.80), for French (L2), *t*(46.02) = 3.54, *p* < 0.001 (one-tailed), *d* = 0.14 (*d_Z_* = 0.55), for German (L1), *t*(30.30) = 3.50, *p* < 0.001 (one-tailed), *d* = 0.16 (*d_Z_* = 0.59), for German (L2), respectively (see Figure 1B).

#### 3.2.2 Accuracies

For accuracies, a lmm analysis using the R function glmer (family = “binomial”) corresponding to the main analysis of RTs was conducted. There were significant main effects of task language, *β* = 0.205, *z* = 2.18, *p* = 0.029; the probability of a correct response was increased for L1 compared to L2. The main semantic priming effect was significant, *β* = 0.236, *z* = 2.69, *p* = 0.004 (one-tailed); it conforms to the RT effect, that is, subsequent to related primes, the probability of correct responses is increased compared to trials with unrelated primes. Thus, there is no indication of a speed-accuracy tradeoff. The priming effect was not moderated by task language (L1 vs. L2), |*z*| < 1. All other effects were not significant, |*z*| < 1.41, except *β* = −0.171, *z* = 1.67, *p* = 0.096 for L1 (German vs. French); error probability tended to be larger for the German sample.

## 4 Discussion

In this study, we recruited French and German sequential bilinguals. The samples successfully passed a test for L2 proficiency in a semantic priming task: Both samples showed significant priming effects in L1 and L2. Numerically, the effect was even stronger in L2 compared to L1. There were no hints to differences between languages (i.e., French vs. German) or samples.^7^

Thus, the sample was ideal for answering the question: Do sequential bilinguals demonstrate effects of embodied language processing in both their first and second language within the modality switch paradigm? Our analyses indeed revealed a significant main effect of modality switch but because of the observed interactions with task language, the answer to this main question is a bit more complex than initially thought.

If the main research question is understood as “Is there *any* evidence for embodied language processing, …, in L2?,” our study yields one clear affirmative answer: For the French language, the medium-sized modality switch effect in L1 (i.e., in the French sample) is accompanied by a significant small-to-medium-sized modality switch effect in L2 (i.e., in the German sample), with no significant difference between the two samples. Of course, for such a between-participants comparison the power was low; therefore, we should not put too much emphasis on this null effect. Nevertheless, if we focus on the French language, we can conclude that embodied language processing, as assessed by the modality switch paradigm, was observed in L2.

For the German language, however, our study yields a different response: We replicated the modality switch effect in L1 (i.e., in the German sample); there was, however, not the slightest evidence of a modality switch effect in L2 (i.e., in the French sample). However, instead of labeling our results inconclusive, we would like to point toward the relevance of an unexpected moderation of switch effects by language. That is, the overall modality switch effect (i.e., irrespective of whether it is an L1 or L2 effect) was tended to be stronger in French than in German. When comparing L1 effects only, it was descriptively stronger in the French than in the German sample (i.e., *d_Z_* = 0.47 compared to *d_Z_* = 0.27; *p* = 0.099). This general language difference appears particularly relevant when interpreting switch effects in L2, given the additional challenge posed by the potentially noisier L2 data resulting from the reduced number of trials included in the analyses (because of lower accuracy in L2): If we accept that L2 effects should be somewhat smaller than L1 effects due to more noisy data, a German L2 effect might simply not be detectable with the limited test-power of the reduced trial number and limited number of participants.^8^

Thus, unexpectedly our study raises a new question: Do languages differ in the magnitude of the modality switch effect and thus in the degree of involuntary sensorimotor processing? It is beyond the scope of the present article (and the expertise of the authors) to speculate about such differences. However, for those readers who feel challenged to think about this issue, the following observation may provide further insight: in Appendix Table A1, we listed studies that used the verification task with the modality switch design in different language samples. Of course, studies differed on more features than only the language, thus, the following observation should be received with some caution. Nevertheless, it is striking that the list can be split (non-overlapping) into four studies using Germanic languages with an average *d_Z_* of 0.30 and four studies using Romanic languages with an average *d_Z_* of 0.63.

Returning to the questions of Kühne and Gianelli (2019), we propose to reject the hypothesis that sensorimotor processes in language processing are restricted to L1—on the basis of our results for the French language.

In general, our experiment was not designed to test competing theories of bilingual language processing, as, for example, the Revised Hierarchical Model (RHM; Kroll and Stewart, 1994) or the (revised) Bilingual Interactive Activation model (BIA+; Dijkstra and Van Heuven, 2002). These theories have great merit in explaining specific effects in (single) word processing. For example, they address the question of whether there are separate lexical stores for L1 and L2 (RHM) or whether there is an integrated lexicon (BIA+; for a discussion, see Brysbaert and Duyck, 2010; Kroll et al., 2010). Sensorimotor representations, however, are not addressed in the models. We nevertheless see two possibilities to relate our research question to these theories.

First, one may generally deny a functional role of sensorimotor processes in language processing. Instead, one may see sensorimotor processes merely as a non-functional rudiment of the L1 learning process which are still triggered in the adult L1 user if a certain word is activated in the L1 *lexicon*. If, by contrast, later L2 learning is based on creating amodal symbolic representations as one may deduct from the assumption of word-concept links in L2 in the RHM, one may expect no modality switch effects in L2 in sequential bilinguals because language processing is based on links between the L2 lexicon and the “concepts” subsystem. Such assumptions, however, would not be supported by our results, given that we do find significant modality switch effects indicating involvement of sensorimotor processing in L2—at least in the German-French bilingual participants.

Second, as alternative, seemingly more plausible location for sensorimotor processes in language processing one may refer to the sub-modules named “concepts” in the RHM or “semantics” in the BIA+. These modules do not distinguish between L1 and L2. Thus, from this perspective there is no room to conceptualize L1/L2 differences in sensorimotor representations in RHM or BIA+ and one would have expected modality switch effects of comparable size in both L1 and L2.

Another theoretical perspective that may be more fruitful with regard to our research questions is given by Yee and Thompson-Schill (2016), who made a strong case for context-dependent conceptual representations. According to this view, conceptual representations are less static than traditionally assumed. On the contrary, “conceptual representations are fluid, changing not only as a function of context as it relates to stimulus modality and task, but also as a function of the context brought by a particular individual— via recent or long-term experience, or even via neural degeneration, processing preferences, or abilities.” (p. 1024).

Transferred to our study: The modality-switch effect in the verification task is typically attributed to the activation of sensorimotor features. However, this does not imply that sensorimotor processing is either necessary or sufficient for task completion. But then again, this does not imply that the commonly observed activation of sensorimotor features is merely an epiphenomenon of language processing. It is one thing to just complete a simple verification task, which may be possible with or without the involvement of sensorimotor processes. It is another thing to have either a rather impoverished, sparse pattern of activated features compared to a rich, embodied pattern activation while processing words in general language comprehension in the context of dynamic interactions with objects and/or social agents. While it may not impact performance in the verification task, it may still be relevant for subsequent thought processes involving the activated concepts.

Applied to our study, the null result for the L2 modality switching effect in French-German bilinguals, may simply signal that such sensorimotor activations are not necessary to complete the verification task. This can also be seen from the difference in magnitude of the L1 effects in German and French that are not accompanied by corresponding differences in the performance of the verification task. However, the results for the French language (irrespective of L1 or L2) indicate that—typically—the verification task is performed with the accompanying phenomenon of sensorimotor activations. This perspective opens a path for future research, away from fundamental discussions whether or not there is something like embodied cognition (see, e.g., Glenberg, 2015; Mahon, 2015a; Mahon, 2015b) to a differentiation if and under what conditions modal and amodal representations play a what kind of functional roles (Kaup et al., 2024; Yee and Thompson-Schill, 2016).

## 5 Limitations

Of course, the interpretability of our current research is limited to the results of one single behavioral experiment, involving limited statistical power for small effects and potentials of false positives or false negatives. Furthermore, our conclusions are only valid to the extent that readers consider the modality switching effect to be a valid indicator of involuntary sensorimotor processes. Pecher et al. (2003) had originally raised a possible “devil’s advocate” criticism that the modality switch effect may be caused by semantic priming processes only. For example, if we assume (a) that the a property of trial *n* − 1 facilitates access to a property of trial *n* if the two properties are semantically related and (b) that properties of the same modality are, on average, semantically more closely related than properties of different modalities, the modality switch effect could be explained as a variant of a semantic priming effect. Pecher et al. (2003) provided an empirical test of both assumptions: In their Experiment 2, they tested whether associatively related properties produce a semantic priming effect in the verification task. The result was a clear null effect, thus lending no empirical support to assumption (a). Moreover, in the same experiment, the modality switching effect was replicated with same-modality pairs where the associative strength between the properties was 0, thus further refuting assumption (b). To our knowledge, no alternative explanation of modality switch effects besides the involvement of sensorimotor activations have been proposed, thus strengthening our assumption that the verification task is a useful experimental paradigm to investigate the role of embodied cognition. Nevertheless, it would certainly be useful to use further paradigms or methods (e.g., transcranial magnetic stimulation; see, e.g., Pobric et al., 2010; Pulvermüller et al., 2005).

## 6 Conclusion

For the French language, we found clear modality switch effects in L1 and L2, supporting the assumption L2 processing does indeed involve sensorimotor processes. However, on the basis of our results for the German language, we can call into question the hypothesis that sensorimotor processes are obligatory in language processing. There was only a meager modality switch effect in L1 that was not accompanied by a performance decline in the verification task. Moreover, a L2 effect was entirely missing. Future research and theorizing should aim to elucidate differences of language processing with and (almost) without accompanying sensorimotor processes.

## Data Availability

The datasets presented in this study can be found in online repositories. The names of the repository/repositories and accession number(s) can be found at: https://osf.io/anzr3.

## Footnotes

^1^For the sake of completeness, Makris (2015) questioned the validity of the effect found by Marino et al. (2014) and proposed an alternative explanation in terms of attentional processes. Buccino and Marino (2015) defended their view of an embodiment perspective.

^2^Data collection also included additional *n* = 15 participants who *a priori* self-categorized as *simultaneous* bilinguals. We invited them for participation for exploratory reasons. Given the limited test power, however, no meaningful analyses can be conducted.

^3^One 15-year old girl participated with parental consent; the remaining age range was 19–26  years.

^4^For example, the original pair “cranberries – tart” was changed into “burger – savory” because the adjectives “tart” and “sour” (used with “buttermilk”) both translate to the German word “sauer.”

^5^Pecher et al. (2003) presented the words “can be” between the noun and the property as a permanent reminder of the task. In German, two different auxiliary verbs would have to be used for the motor modality (e.g., RASENMÄHER kann GESCHOBEN *werden*. [LAWNMOWER can be PUSHED]) compared to the other modalities (e.g., SALSA kann SCHARF *sein* [SALSA can be SPICY]). In order to avoid confounding, we did not present the analog of “can be” in each trial, of course for both the German and French blocks.

^6^In line with the analyses of RTs, we discarded trials with an error response in the context trial.

^7^As an aside it might be noted that the L2 effect was more pronounced for those participants who started by L1. A similar cross-language effect was recently found by Norman and Peleg (2022).

^8^The following thought experiment may help to put our results into perspective. Imagine that we would have conducted two L1 pilot studies, one with native speakers of French, one with native speakers of German, observing the L1 effects that we found in our actual study: One substantial modality switch effect in French, one meager, just significant effect in German. We would have of course continued with a main study that would have focused on the French language (i.e., would have compared a sample of French participants [L1] with a sample of German participant [L2]), simply because the corresponding study with regard to the German language would have been too costly (in terms of participants needed). In the report of the pilots and the main study we would have noted (a) that it is up to further research to explore whether there are really differences in (L1) modality switch effects for different languages (but that it is beyond the scope of the present article to elaborate on this issue). But (b), we would have noted that for a language that produced a clear L1 modality switch effect, a corresponding L2 modality switch effect can be found as well. The factual situation is not very different. The only thing is that we did not knew in advance that the German (L1) modality switch effect would be so small. Therefore, we collected corresponding L2 data although this part was – now seen from a post-hoc perspective – totally underpowered.

## Appendix

**Table A1:** Effect sizes of the modality switch effect (all L1) in different studies.

Study	Language	*d_Z_*
van Dantzig et al. (2008)	Dutch	0.27
*This study*, German sample	German	0.27
Pecher et al. (2003), Exp. 1	English	0.33
Pecher et al. (2003), Exp. 2	English	0.33
Marques (2006), Exp. 1	Portugese	0.37
*This study*, French sample	French	0.47
Scerrati et al. (2015)	Italian	0.71
Vermeulen et al. (2007)	French	0.98
